# Insight into Cd Detoxification and Accumulation in Wheat by Foliar Application of Ferulic Acid

**DOI:** 10.3390/plants14101436

**Published:** 2025-05-11

**Authors:** Simeng Li, Wenyang Fu, Bingling Li, Yi Wang, Yiran Cheng, Houyang Kang, Jian Zeng

**Affiliations:** 1 College of Resources and Science Technology, Sichuan Agricultural University, Chengdu 611130, China; lisimeng@stu.sicau.edu.cn (S.L.); wyang_08@163.com (W.F.); rosinking@163.com (B.L.); 2 Triticeae Research Institute, Sichuan Agricultural University, Chengdu 611130, China; wangyi@sicau.edu.cn (Y.W.);; 3 State Key Laboratory of Crop Gene Exploration and Utilization in Southwest China, Sichuan Agricultural University, Chengdu 611130, China

**Keywords:** ferulic acid, Cd detoxification, tissue distribution, root retention, Cd transporter gene regulation

## Abstract

Cadmium (Cd) contamination in agricultural soils poses a significant threat to human health through the food chain. It is of great significance to address safe wheat production in Cd-contaminated agricultural soils. This study employed foliar spraying of ferulic acid (FA) in both hydroponic and field trials to investigate its potential in alleviating Cd toxicity and reducing Cd accumulation in wheat grains. Our findings revealed that FA application at 20 and 50 μM promoted plant growth, increased photosynthetic efficiency, and enhanced root tolerance to Cd by increasing mean root diameter, surface area, and root tip number, as well as enhancing antioxidant defense in roots. Especially, 20 μM FA foliar application significantly alleviated Cd-induced growth inhibition in seedlings and reduced grain Cd content by 66.3% compared to Cd-stressed alone. Mechanistically, FA downregulated the Cd transporter gene *TaHAM2* to reduce Cd translocation from roots to shoots, while upregulated the Cd cellular compartment gene *TaHAM3* to increase root Cd retention, of which 82.9% was sequestered in roots. During the grain-filling period in the field trial, FA application reduced Cd transport from roots to stems and stems to rachides, but enhanced Cd retention in rachides and roots. Additionally, FA downregulated the phloem Cd loading gene *LCT1*, limiting Cd allocation to bracts and grains, which in turn lowered the Cd content in the grains. Collectively, FA foliar application modulated Cd transport pathways by coordinately downregulating xylem and phloem transporter genes and enhancing root Cd retention capacity. These findings established FA as a promising strategy for Cd detoxification and reduced accumulation in crop grains through integrated physiological and molecular interventions. Overall, it holds potential for the future development of safe crop production in soils polluted with Cd.

## 1. Introduction

With the intensification of industrialization and human activities, the bioavailability of cadmium (Cd) in the environment has continued to rise, posing severe threats to agricultural productivity and human health. As one of the most toxic heavy metals, Cd can be absorbed by plant roots and biomagnified through the food chain, ultimately leading to renal dysfunction, osteoporosis, and other diseases [1]. Under Cd stress, plants experience intracellular reactive oxygen species (ROS) bursts, triggering protein degradation, membrane lipid peroxidation, and even programmed cell death [2,3]. In China, the limiting threshold for Cd in crop grains is less than 0.1 mg kg^−1^ according to the standard of the maximum limit of contaminants in food (GB 2762-2022) [4]. Recent data indicate 21.49% of agricultural soils in major grain-producing regions surpass Cd safety limits, leading to annual contamination of 12 million metric tons of crops and economic losses exceeding 20 billion CNY [5]. Therefore, developing strategies for safe crop production in Cd-contaminated farmland is urgently needed.

Cd uptake occurs by apoplast and symplast pathways using various ion channels and metal transporters in roots, followed by further translocation to edible parts through the xylem and phloem [6]. For instance, several plasma membrane transporters for Cd uptake, sequestration, and transportation have been identified [7]. The low-affinity cation transporter *LCT1* has been reported to be involved in Cd loading in the phloem, which reduces the transport of Cd from xylem to phloem and lowers the Cd concentration in phloem sap by silencing its expression [8,9]. The members of heavy metal ATPase (HMA) family facilitate the compartmentalization of Cd into the vacuole and mediate long-distance transport from roots to stems [10,11]. Subcellular distribution analyses reveal that 53–72% of Cd is immobilized in cell walls, 18–44% is complexed with thiol ligands (e.g., phytochelatins) in vacuoles, and less than 13% enters organelles [12]. However, unsequestered Cd can still migrate to grains via transpiration-driven flow, compromising food safety.

Ferulic acid (FA), a ubiquitous phenolic compound, exhibited significant potential in enhancing plant stress resistance due to its antioxidant properties and ability to modulate cell wall cross-linking [13,14]. The existence of antioxidant properties through its conjugated side chains that readily form stable phenoxy groups enabled effective scavenging excessive ROS accumulation induced by adversity, especially under heavy metal stress [15]. FA formed covalent cross-links with cell wall polysaccharides to form structural complexes that immobilized Cd ions in the cell wall, limiting translocation and thus enhancing plant tolerance to Cd [16]. Recent studies have identified the protective function of phenolic acids in mitigating Cd toxicity in tobacco and rice [17,18]. These studies mechanistically demonstrated that phenolic acids limit Cd transport from roots to shoots by up-regulating metal transporter genes such as *HMA2* and *NRAMP5*. Therefore, FA application to crop safety production has potential under Cd contamination fields. However, the regulatory effects of FA on Cd uptake and accumulation as well as Cd-associated transporters in wheat remain underexplored.

Foliar barrier agents as a kind of effective solution, enter into the plant through the cuticle and stomata of leaf epidermal cells, and can affect the migration and redistribution of heavy metals in plant tissue by competing for transport channels, modulating nutrient partitioning, and enhancing antioxidant capacity [19,20]. Foliar barrier technology has emerged as a promising strategy to reduce grain Cd accumulation. The grain-filling period is critical for Cd accumulation in wheat, with approximately 60% of grain Cd derived from root uptake and 40% from redistribution of vegetative organs [21]. During the grain-filling stage, plant roots continue to take up Cd from the soil and transport it through the xylem to the aboveground tissues and directly to the grains, where it is also redistributed to the grain from other organs through the phloem [22]. It was shown that Cd uptake by roots and Cd redistribution by vegetative organs are equally important for Cd accumulation in wheat grain [23]. The study of Cd accumulation and distribution in various organs in wheat plants revealed that the glumes had a good retention effect on Cd and affected the accumulation of Cd in the grain [24]. It is well known that transpiration pull plays an important role in the transport process of heavy metals [25]. Meanwhile, Cd accumulation in the spike is not mainly allocated to the grains but migrates and accumulates in high-transpiration organs of bracts [21].

We hypothesize that foliar FA application affects the expression of Cd-related transporter genes and regulates redistributive effects by fixing Cd and inhibiting Cd transport in wheat tissues. To address this hypothesis, this study integrated hydroponic and field experiments to investigate the alleviative effects of foliar FA application on Cd toxicity and its regulatory mechanisms on Cd accumulation in wheat. We aim to (1) elucidate the role of FA in remodeling root morphology and photosynthetic efficiency under Cd stress; (2) decipher FA-mediated Cd subcellular compartmentalization and expression patterns of transporter genes; and (3) unravel dynamic Cd allocation patterns in plant during the grain-filling period. The findings will provide theoretical and technical foundations for safe wheat production in Cd-contaminated environments.

## 2. Materials and Methods

### 2.1. Plant Material and Growth Conditions

Seeds of common wheat variety (Chuanmai 104) developed by Sichuan Academy of Agricultural Sciences were sterilized with H_2_O_2_ (10%, *v*/*v*) for 10 min and rinsed in distilled water for 12 h, and then germinated on moist filter paper at room temperature for 4 days. At three-leaf stage, the wheat seedlings with uniform growth vigor were transplanted into the hydroponic pot filled with Hoagland’s solution. An original control group without Cd stress and FA foliar application was established. The treatments of FA foliar under different Cd conditions were as follows: 50 μM Cd + 0 μM FA; 50 μM Cd + 20 μM FA; 50 μM Cd + 50 μM FA; 50 μM Cd + 100 μM FA; 20 μM FA + 0 μM Cd; 50 μM FA + 0 μM Cd and 100 μM FA + 0 μM Cd. The FA solution was sprayed on both adaxial and abaxial surfaces of the leaves every 7 d for 14 d until dripping occurred. The final doses were approximately 150 μL per plant. The seedlings were grown in a growth chamber with 500 µmol m^−2^ s^−1^ photosynthetic photon flux density, 70% relative humidity, a 14/10 h light/dark cycle for 2 weeks. Plant samples from each treatment group were divided into roots and shoots. Some samples were immediately dried at 70 °C to achieve constant weight as plant biomass, and the rest of the fresh samples were stored at −80 °C for subsequent physiological and biochemical analyses.

Initial hydroponics experiment revealed that wheat plant exposed to Cd stress exhibited the optimal growth performance when treated with 20 μM FA application. Therefore, the field trials employed foliar applications of 20 μM FA to assess its agronomic suitability. The field experiment was conducted at the experimental station of Sichuan Agricultural University (103°51′ E, 30°42′ N). Prior to treatment, the soil basic physicochemical properties were quantified as follows: pH 6.32, available nitrogen of 60.2 mg kg^−1^, available phosphorus of 67.1 mg kg^−1^, available potassium of 80.4 mg kg^−1^, total Cd content of 0.36 mg kg^−1^. The experiment was arranged in randomized blocks with two treatments and three biological replicates. The sowing density of each plot (2.7 m^2^) was about 200 wheat plants: (1) control (CK, foliar spray with deionized water); and (2) FA application (20 μM FA solution applied foliarly during the grain-filling stage). Foliar applications were administered at 7-day intervals after flowering, with each application occurring between 4 and 5 p.m. The amount of foliar spray solution was 150 mL per plot to achieve full wheat foliar coverage until dripping occurred. The first sampling was conducted after 7 days. Plants were harvested at physiological maturity following four consecutive applications, with five sampling events conducted throughout the experimental period. All the harvested samples were separated into eight tissue parts of roots, stems, leaves, glumes, bracts, grains, awns, and rachides and subsequently oven-dried at 70 °C until constant weight was achieved, followed by homogenization for biochemical and elemental analyses.

### 2.2. Root Morphological Investigation

The intact root system was digitally scanned using an Epson perfection scanner (Epson V850, Epson Co., Ltd., Beijing, China) to produce high-quality images. Quantitative root morphological parameters, including total root length, root surface area, root volume, root average diameter and root tip number were analyzed using the LA-S root analysis system (Wanshen Detection Technology Co., Ltd., Hangzhou, China).

### 2.3. Photosynthesis Assay

Gas exchange parameters were measured for the uppermost, fully expanded leaf in randomly chosen individuals from each treatment between 9:00–12:00 am using a LI-6400 portable photosynthesis system (LI-COR, Lincoln, NE, USA). Leaves were allowed to equilibrate for 5 min at 500 µmol m^−2^ s^−1^ photosynthetic photon flux density before measurements. The CO_2_ concentration in the cuvette during the measurements was 400 ± 5 µmol mol^−1^, and the relative humidity was at 65% inside the cuvette. Once the steady-state gas exchange rates (<5% coefficient of variation over 3 min) were observed at these conditions, net photosynthetic rate (*P*_n_), stomatal conductance (*g*_s_), transpiration rate (*T*_r_), and intercellular CO_2_ concentration (*C*_i_) were recorded.

### 2.4. Biochemical Traits Assays

Malondialdehyde (MDA) content was determined according to the method of Hodges et al. (1999) [26]. About 1.0 g of fresh sample was homogenized in 10 mL of 10% (*w*/*v*) trichloroacetic acid (TCA) and centrifuged at 12,000 rmp for 10 min, after which 2 mL of supernatant was mixed with 2 mL of 0.6% thiobarbituric acid (TBA) and heated at 95 °C for 15 min and then quickly cooled down on ice. After centrifugation at 12,000 rmp for 10 min, the absorbance of the supernatant was determined at 450, 532, and 600 nm. The MDA content (nmol mg^−1^) = [6.45 × (A_532_ − A_600_) − 0.56 × A_450_] × V_t_/W, where V_t_ was the extraction solution volume, and W was the sample quality.

About 1.0 g of fresh sample was added into 5 mL precooled 0.05 mol L^−1^ PBS solution, homogenized in an ice water bath, and centrifuged at 4 °C 12,000 rpm for 10 min and then the supernatant was prepared. Antioxidant enzyme activities, including superoxide dismutase (SOD), peroxidase (POD) and catalase (CAT) were quantified using commercial assay kit (Kemin Biotechnology Co., Ltd., Suzhou, China) according to the standardized protocols. Specifically, the activities of SOD, CAT and POD were determined using the SOD assay kit (SOD-1-W), CAT assay kit (CAT-1-W) and POD assay kit (POD-1-Y), respectively. The POD and SOD activity were expressed as units of enzyme activity per gram of fresh weight, while CAT activity was expressed as μmol min^−1^ g^−1^ of fresh weight.

### 2.5. Determination of Cd Concentration

About 0.1 g of ground samples was digested with mixed acid (HNO_3_/HClO_4_, *v*/*v* = 4:1) at 220 °C for 5 h, sequentially diluted to 50 mL before filtering. The concentration of Cd was determined by inductively coupled plasma mass spectrometry (ICP-MS) using a Nexlon2000 instrument with detection limit of 0.05 μg L^−1^ (PerkinElmer, Waltham, MA, USA).

The determination of subcellular Cd concentration was analyzed based on the methods of Weigel (1980) and Gabbrielli et al. (1990), with modifications [27,28]. Briefly, about 1.0 g of fresh tissues was homogenized with the extraction buffer (0.25 M sucrose + 1 mM dithioerythritol + 50 mM Tris-HCl, pH 7.5) to produce a cell wall fraction (F_cw_) by centrifugation at 3000 rpm for 15 min, a cell organelle fraction (F_co_), and a soluble fraction (F_s_) by centrifugation at 10,000 rpm for 30 min. These fractions were then dried and digested with HNO_3_-HClO_4_ (*v*/*v* = 4:1) mixed acid at 220 °C for 5 h and diluted 25 mL before filtering. The Cd concentration in each fraction was determined by ICP-MS.

### 2.6. Cd Uptake and Translocation Related Gene Quantification

Fresh seedling roots, stems, and leaf tissue were used for total RNA extraction using the RNeasy Plant RNA Mini Kit (Qiagen, Hilden, Germany), followed by genomic DNA elimination and reverse transcription to synthesize cDNA using the PrimeScript™ FAST RT Reagent Kit with gDNA eraser (Takara Biotechnology Co. Ltd., Dalian, China). Four genes related to Cd uptake and transfer were screened to investigate the expression in plant tissues. *TaNramp5* encoded Cd influx transporter facilitating the Cd from soil to roots, *TaHAM2* and *TaHAM3* encode transporter proteins mediating root-to-shoot translocation, and *TaLCT1* encoded Cd transporter involved in Cd transport to grains were quantified. The qRT-PCR amplification procedure was performed as follows: 95 °C for 2 min, 95 °C for 10 s, 60 °C for 10 s, 72 °C for 15 s, and melting for 6 s, all for 40 cycles. The relative gene transcript levels were calculated using the 2^−ΔΔCt^ method with the internal control gene *TaActin* as a reference. The primers for RT-PCR were listed in Appendix A, Table A1.

### 2.7. Data Analysis

Translocation factors (TFs) were calculated to estimate the plant’s capacity for metal partitioning between different tissues, as defined by the following formula: TF_n/m_ =$\;\frac{C_{m}}{C_{n}}$, where C represents the Cd concentration (mg kg^−1^) in the recipient tissue m and donor tissue n, respectively. The grain-filling rate and grain Cd accumulation rate were characterized using established methodologies [3], with the grain-filling rate equal to $\frac{\Delta m}{\Delta t}$, where m denotes the 1000-grain weight (mg) at specific phenological stages, and Δm represents the incremental weight grain between consecutive sampling intervals (Δt, days). The grain Cd accumulation rate equals $\frac{\Delta n}{\Delta t}$. This parameter is derived from the following relationship: n=m × c, where n indicates the total Cd content per 1000 grains (mg), *c* represents the grain Cd concentration (mg kg^−1^) at corresponding stages, and Δn reflects Cd content increments between adjacent sampling periods.

All the data are presented as the mean ± standard deviation, and statistical significance was determined through one way analysis of variance (ANOVA), followed by Duncan’s tests for multiple comparisons. The threshold for statistical significance was set to *p* < 0.05 for all analyses. All data were analyzed using IBM SPSS Statistics 28.0 (SPSS Inc., Chicago, IL, USA). Plotting was performed using OriginPro 2024 (OriginLab Corporation, Northampton, MA, USA).

## 3. Results

### 3.1. Plants Growth Performance

Exogenous foliar FA application demonstrated regulatory effects on wheat seedling growth, exhibiting growth promotion and Cd stress-specific morphological adaptions (Table 1). While FA significantly enhanced biomass accumulation in both roots and shoots without Cd stress, its effects under Cd stress displayed tissue specificity. Root development showed statistically significant improvements. Notably, FA induced modifications in the root–shoot allocation patterns of biomass under Cd stress, as evidenced by a 23.0% increase in root biomass accumulation and a 22.2% elevation in the root-to-shoot ratio under 20 μM FA application compared to Cd stress alone.

By analyzing root morphological indicators, it was found that exogenous FA significantly influenced root development in wheat with or without Cd exposure (Table 2). The FA treatment significantly enhanced several key root morphological traits, including root surface area, root volume, average root diameter, and root tip number when compared to the controls. Moreover, FA at 100 μM was the most effective in altering the root morphology among the various treatment concentrations. Compared with those treated with Cd alone, the conditions of 50 and 20 μM FA combined with Cd significantly increased root volume and average root diameter. Furthermore, the application of 20 μM FA led to a significant increase in root surface area. However, root elongation was significantly inhibited by Cd exposure combined with 50 and 100 μM FA. Therefore, FA application at 20 μM had a significant mitigation effect on root growth inhibition under Cd stress.

### 3.2. Effect of FA on Photosynthesis

**Figure 1:**
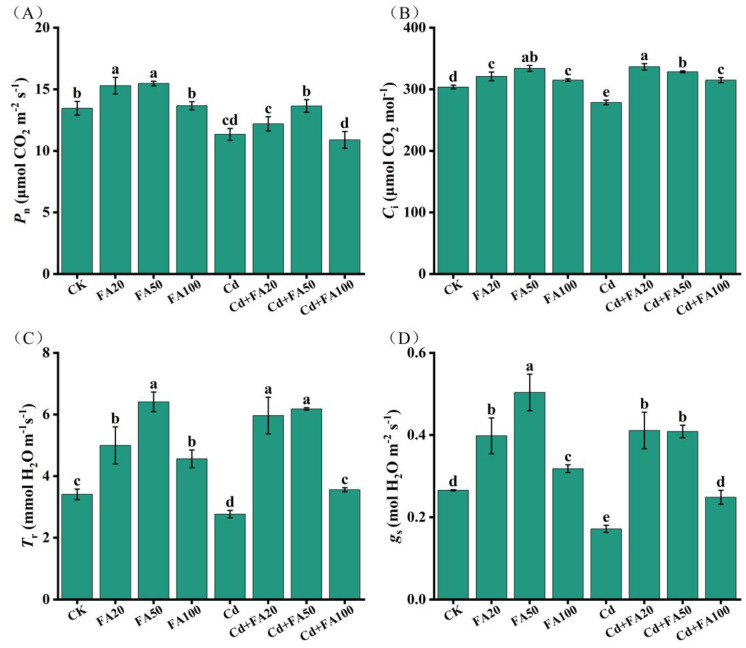
Effects of ferulic acid on photosynthesis in wheat seedlings. (**A**) Net photosynthetic rate (*P*_n_); (**B**) intercellular CO_2_ concentration (*C*_i_); (**C**) stomatal conductance (*g*_s_); (**D**) transpiration rate (*T*_r_). All data are presented as the mean ± standard deviation of three biological replicates. Different lowercase letters indicate significant differences between treatments (*p* < 0.05).

Foliar FA application significantly enhanced photosynthetic performance in wheat seedlings, with differential modulation patterns between control and Cd-stressed conditions (Figure 1A–D). Wheat plants treated with FA alone exhibited dose-dependent increases in photosynthetic parameters, with the most significant effect observed at 50 μM FA, where *P*_n_, *C*_i_, *g*_s_ and *T*_r_ increased by 14.9%, 10.2%, 89.8% and 87.9%, respectively. Under Cd stress, *C*_i_, *g*_s_ and *T*_r_ were significantly affected by different concentrations of FA application, while *P*_n_ showed a significant improvement only at 50 μM FA (Figure 1A). Notably, 20 and 50 μM FA treatments exhibited superior efficacy in ameliorating photosynthetic performance compared to 100 μM FA. Relative to plants stressed with Cd alone, the 20 and 50 μM FA treatments resulted in a 141.2% and 139.6% increase in *g*_s_, increased *C*_i_ by 20.8% and 17.9%, respectively, and elevated *T*_r_ by 120.7% and 114.2% (Figure 1C,D).

### 3.3. Effect of FA on Antioxidant Defense Capacity

**Figure 2:**
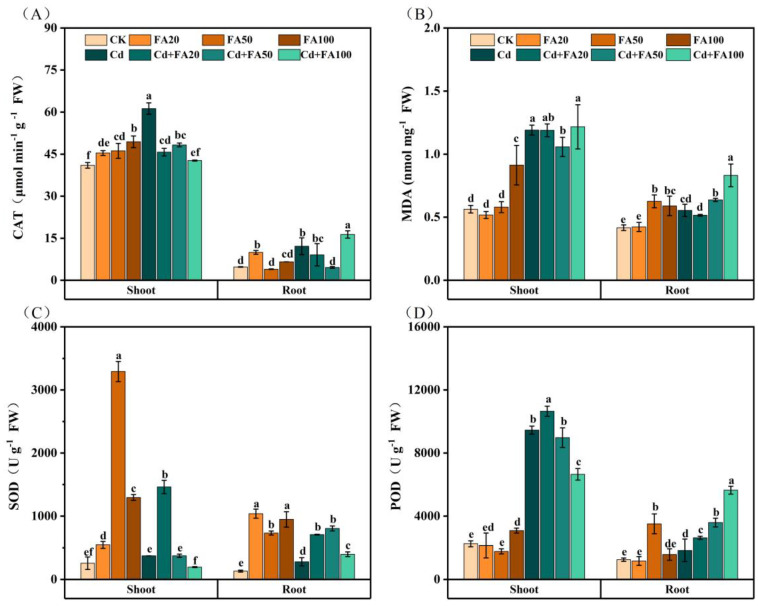
Effects of ferulic acid on antioxidant enzyme activities in the shoots and roots of wheat seedlings with Cd or without Cd exposure. (**A**) Catalase (CAT); (**B**) malondialdehyde (MDA); (**C**) superoxide dismutase (SOD); (**D**) peroxidase (POD). All data are presented as the mean ± standard deviation of three biological replicates. Different lowercase letters indicate significant differences between treatments (*p* < 0.05).

Compared with the control, foliar FA application induced a significant increase in CAT activity in the shoots by 38.4–106.2%, whereas root CAT activity showed concentration specificity, with only the 20 μM FA application causing a significant increase of 60.1%. However, FA treatment paradoxically reduced CAT activity by 26.9–165.2%, except at 100 μM, where CAT activity in the roots showed a 34.4% recovery under Cd exposure (Figure 2A). FA application at 20 μM had no effect on the MDA content, but 100 μM FA elevated root and shoot MDA by 40.5% and 61.9%, respectively, compared to the control. Under Cd stress, FA application preferentially enhanced root MDA without affecting shoot MDA accumulation (Figure 2B). SOD activity showed a significant dose-dependent increase, with 20–100 μM FA application increasing SOD activity in the roots by a factor of six to eight and in the shoots by a factor of two to thirteen. However, FA application alone significantly increased POD activity in the shoots and roots only at 100 and 50 μM concentrations, respectively. FA was effective in modulating the antioxidant defense system in the roots under Cd-exposed conditions, increasing SOD and POD activities by 42.5–187.5% and 42.7–206.9%, respectively, while the response of the shoots showed concentration-selective modulation, with only 20 μM of FA significantly increasing SOD (289.8%) and POD (12.7%) activities (Figure 2C,D).

### 3.4. Effect of FA on Cd Subcellular Distribution and Translocation

**Figure 3:**
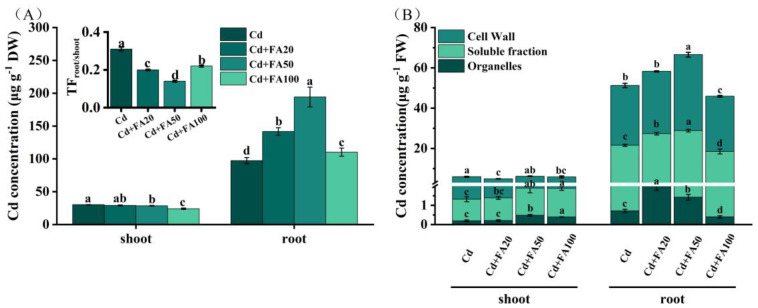
Effects of ferulic acid on tissue (**A**) and subcellular (**B**) Cd concentrations in wheat seedlings under Cd stress. All data are presented as the mean ± standard deviation of three biological replicates. Different lowercase letters indicate significant differences between treatments (*p* < 0.05).

Root Cd accumulation was significantly enhanced by FA application, while the shoot Cd level decreased inversely at 50 and 100 μM FA compared to Cd stress alone (Figure 3A). FA application significantly reduced the root-to-shoot TF by 38.5–121.4% compared to Cd stress alone, with the most effective concentration being 50 μM FA. Additionally, FA application preferentially influenced the subcellular distribution of Cd in roots, as evidenced by a significant increase in Cd content in the soluble fraction and in the organelles in the roots (Figure 3B). At 20 and 50 μM FA, the Cd concentration in the soluble fraction increased by 21.46% and 31.3%, and in the organelles by 153.5% and 102.8%, respectively.

### 3.5. Expression Patterns of Genes

**Figure 4:**
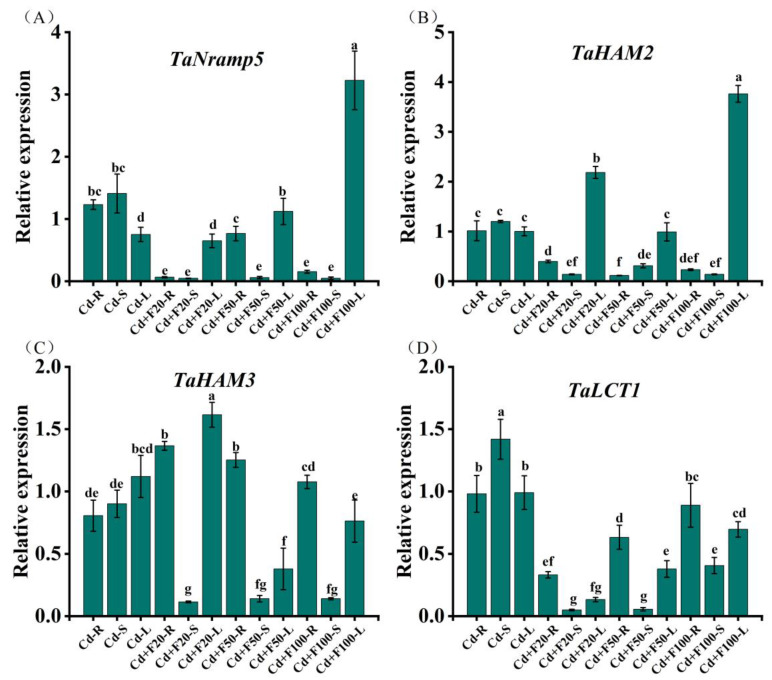
Expression levels of *TaNramp5* (**A**), *TaHAM2* (**B**), *TaHAM3* (**C**) and *TaLCT1* (**D**) in roots (R), stems (S), and leaves (L) of wheat seedlings under different treatments. Data are presented as the mean ± standard deviation of three biological replicates. Different lowercase letters indicate significant differences between treatments (*p* < 0.05).

Foliar FA application induced significant transcriptional regulation of Cd transport and accumulation-related genes in wheat seedlings under Cd stress, compared to Cd stress alone. Specifically, we observed the marked downregulation of the *TaNramp5* gene, which is associated with Cd uptake, and the Cd transporter gene *TaHAM2*, both of which were significantly downregulated in the roots and stems under FA application combined with Cd exposure, compared to Cd exposure alone (Figure 4A,B). In contrast, the vacuolar sequestration-related gene *TaHAM3* showed significant upregulation in the roots and leaves, only at 20 μM FA combined with Cd exposure (Figure 4C). Furthermore, the phloem loading-associated transporter gene *TaLCT1* exhibited pronounced suppression in the roots, stems, and leaves, particularly under 20 and 50 μM FA application combined with Cd exposure (Figure 4D).

### 3.6. Cd Allocation Dynamics During the Grain-Filling Stage

**Figure 5:**
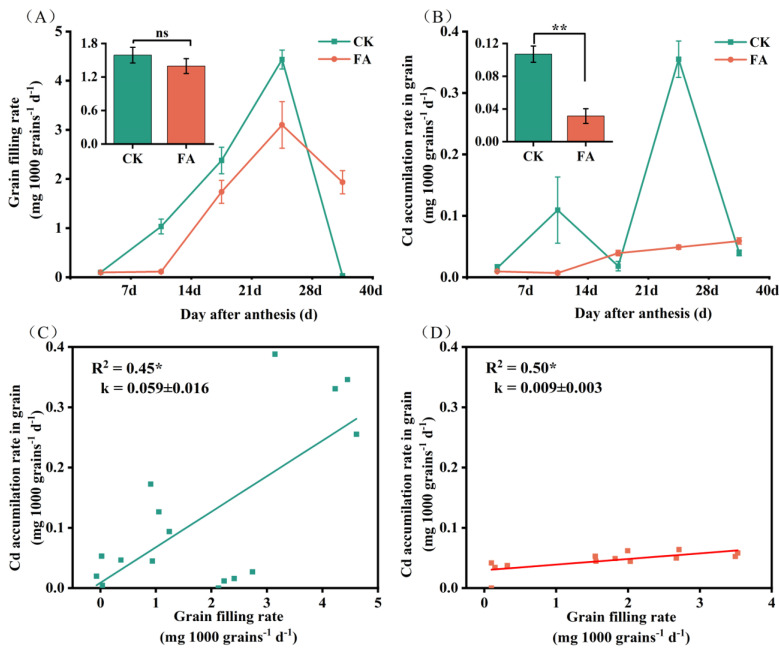
Changes in grain-filling rate and grain Cd accumulation rate during the grain-filling period of wheat and their linear relationships under different treatments. Grain-filling rate (**A**); grain Cd accumulation rate (**B**); linear relationship between the grain-filling rate and the Cd accumulation rate under the CK treatment (**C**) and FA treatment (**D**). The mean values of the grain-filling rate and grain Cd accumulation rate are shown in the upper-left panel of (**A**,**B**). All data are presented as the mean ± standard deviation of three biological replicates. * and ** indicated significant differences between treatments at *p* < 0.05 and *p* < 0.01, respectively. ns indicated no significance.

**Figure 6:**
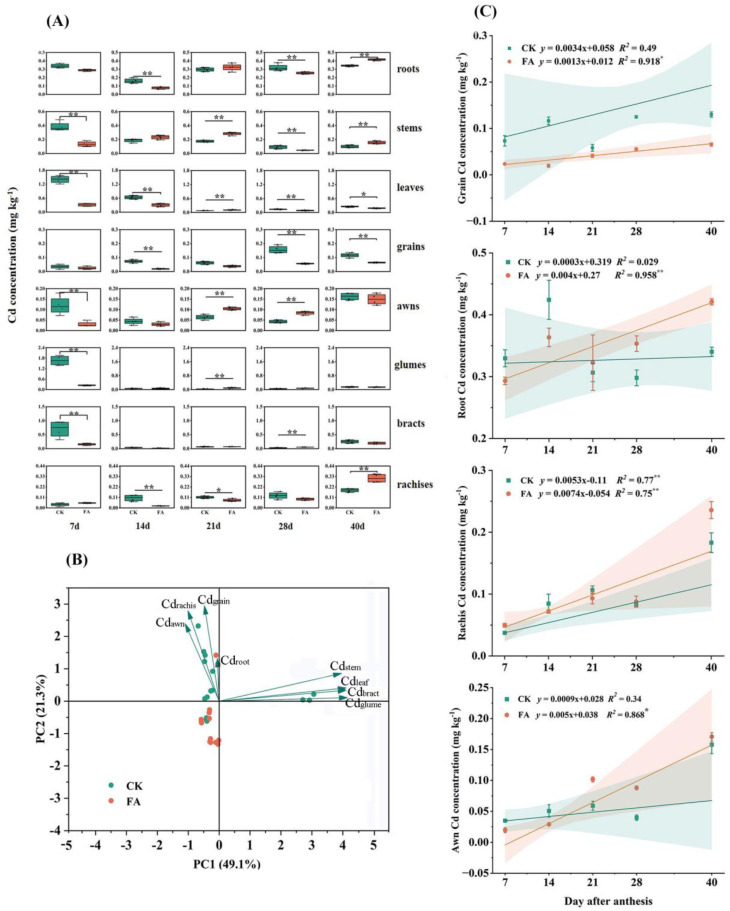
Dynamics of Cd content in different plant tissues during the wheat grain-filling period under CK and FA treatments (**A**); principal component analysis of Cd content in different parts of wheat plants at the filling stage (**B**); linear relationship between grain-filling time and Cd concentration in grains, roots, rachides, and awns (**C**). All data are presented as the mean ± standard deviation of three biological replicates. * and ** indicate significant differences between treatments at *p* < 0.05 and *p* < 0.01, respectively.

**Figure 7:**
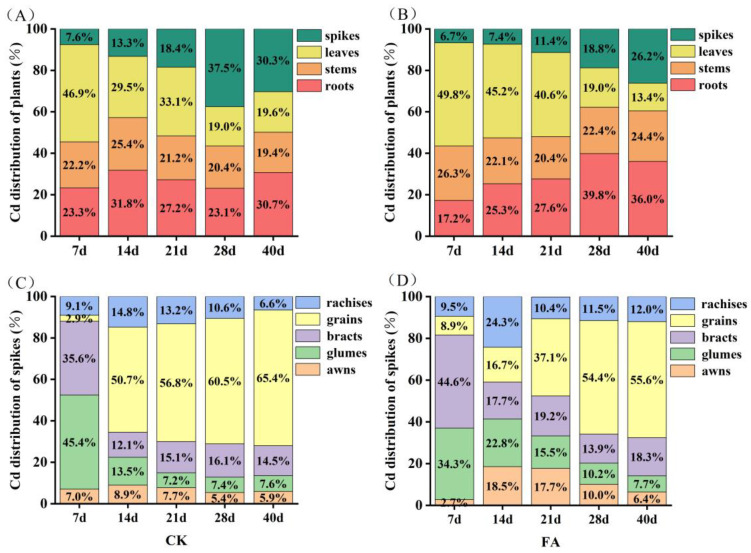
Cd distribution in different wheat plant tissues during the grain-filling period. Changes in Cd proportions in wheat plant tissues under CK (**A**) and FA treatments (**B**). Changes in Cd proportions in spike organs under CK (**C**) and FA treatments (**D**).

During the grain-filling stage, FA application altered Cd translocation dynamics, effectively mitigating grain Cd accumulation (Table 3). FA application significantly reduced Cd root-to-stem translocation factor by 16.0–111.8% compared to the CK treatment, while doubling Cd allocation to leaves. Conversely, stem–rachis transfer decreased by eight-fold, except at 7 days post-anthesis, and Cd flux from rachides to grains decreased progressively, reaching a 110.4% reduction at maturity stage. FA concurrently slowed grain-filling rates by 30.1% and halved Cd accumulation rates relative to Cd stress, with linear regression confirming a two-fold reduction in Cd influx per unit filling rate (Figure 5). Consequently, the FA application lowered final grain Cd content to 66.3% of Cd-stressed plants, maintaining grain Cd content below 0.1 mg kg^−1^, which complies with China’s food safety standards (GB 2762-2022) [4] throughout the grain-filling period (Figure 5D). Under the CK treatment, Cd concentrations in roots, awns, and bracts initially declined and then increased, while Cd concentrations in stems, leaves, and glumes gradually decreased. The Cd concentrations in grains and rachides tended to increase. Tissue-specific Cd dynamics during grain filling revealed that FA treatment preserved tissue Cd accumulation trend under the CK treatment in most tissues but induced distinct Cd fluctuations in stems and sustained Cd accumulation in awns (Figure 6). In addition, compared to CK, the FA treatment significantly reduced Cd concentrations in the corresponding tissues at 7, 14, and 21 days after flowering. At the maturity stage, FA-treated plants exhibited significantly elevated Cd concentrations in roots, stems, and rachides, and reduced Cd concentrations in grains and leaves relative to the CK treatment (Figure 6A). Principal component analysis was performed on the Cd concentrations in different wheat tissues. The degree of interpretation of the first axis was 49.1%, and that of the second axis was 21.3%, yielding an overall degree of interpretation of 70.4%. Cd concentration in roots, rachides and awns had positive effects on grain Cd (Figure 6B). Accordingly, a highly significantly increased in Cd concentration with grain filling time was observed for roots, rachides and awns under FA treatment (Figure 6C). Compared to CK, FA application reduced the Cd distribution in spikes (Figure 7A,B). Within spike tissues, FA application preferentially redirected Cd to non-edible components such as rachides and bracts at maturity stage. In this case, the Cd distribution increased by 81.8% in rachides and 26.2% in bracts in plants treated with FA compared to the CK treatment (Figure 7C, D). It can be speculated that the increase in root Cd, rachis Cd, and bract Cd may be one of the main reasons for the reduction in Cd concentrations in grains.

## 4. Discussion

### 4.1. FA-Mediated Regulation of Cd Partitioning in Plant Tissues

The spatial distribution and translocation dynamics of Cd in crops are fundamentally governed by root absorption and xylem or phloem transport [14]. Our findings demonstrated that FA foliar application exerted multiple regulatory effects on Cd distribution pattern, finally achieving grain Cd concentrations compliant with food safety standards.

Root sequestration forms the primary barrier against Cd upward translocation [29]. Compared to Cd stress alone, FA treatment significantly increased Cd concentrations in roots and accumulated in a time-dependent manner during grain filling with a persistent retention effect. Moreover, the subcellular distribution pattern of Cd indicated that FA-induced preferential Cd allocation to cell walls and soluble components, which was most pronounced in roots. It has been shown that root xylem loading is critical for Cd transport from roots to shoots [30]. Inhibition of Cd loading in the xylem reduced the efficiency of root-to-shoot translocation, which increased root Cd concentrations and reduced Cd mobility, thus causing Cd retention in roots [31]. FA application in this study reduced the root-to-shoots transport coefficient. Therefore, FA-induced Cd immobilization reduced root-to-shoot Cd flux, which was consistent with established mechanisms linking reduced xylem sap Cd content to diminished shoot allocation [32,33]. It was shown that Cd in roots consistently provided 60% of the Cd accumulation in grains in terms of Cd remobilization in wheat tissues [21]. FA treatment significantly immobilized Cd in roots during the grain-filling period and reduced Cd transfer to aerial parts, thus suggesting that FA reduced the remobilization capacity of Cd in roots, which was beneficial in reducing Cd accumulation in grains.

The redistribution dynamics of Cd during grain filling are critical determinants of Cd accumulation in grain. Our findings were consistent with previous inference [34] that Cd accumulation in stems remained relatively stable during the grain-filling period in crops. Notably, FA treatment altered the Cd distribution pattern by shifting the Cd flux towards the leaves and away from the rachis, suggesting modified xylem-to-phloem transfer at critical junctions for Cd partitioning [35]. This reconfiguration implied that FA interfered with Cd mobilization pathways prior to phloem loading, thereby limiting subsequent transfer from rachis to developing grains.

Cd accumulation in wheat spikes demonstrated a temporal progression during grain development, with FA-treated plants exhibiting consistently lower Cd concentrations in spikes compared to controls. Compared with control plants, FA treatment caused a continuous increase in the state of Cd accumulation in the rachides from 21 days after anthesis and caused higher Cd accumulation in the glumes and awns than that under control from 14 days after anthesis. Moreover, a significant increase in Cd concentration of awns with grain filling time was observed under FA treatment. These findings collectively indicated that FA application promoted Cd sequestration within the rachides and restricted its translocation to developing grains. The resultant redistribution pattern enhanced Cd allocation to non-grain spike components while diminishing transfer to grains, thereby effectively reducing grain Cd content under FA treatment.

### 4.2. Potential Mechanism of FA Mediated Cd Mitigation

Cd toxicity in plants is well documented to impair enzymatic activities, photosynthetic efficiency, and light energy utilization, ultimately disrupting metabolic homeostasis and restricting plant development [36]. To counteract Cd stress, plants employ multiple strategies including subcellular compartmentalization, vacuolar sequestration, osmotic adjustment, and antioxidant defense [37]. Our findings confirmed the stress-alleviating properties of FA, demonstrating its capacity to enhance root Cd tolerance and restrict Cd translocation in wheat seedlings, thereby mitigating Cd phytotoxicity.

Consistent with previous reports on FA-induced root growth promotion under environmental stress [38], our results revealed significant FA-mediated improvement in wheat seedling growth under Cd exposure, particularly in root systems. FA treatment induced root morphological trait modifications characterized by increased average diameter and reduced root surface area that likely impeded Cd uptake, given the established correlation between diminished fine root proliferation and reduced Cd absorption [39]. Cd-induced oxidative stress was manifested through ROS production imbalance, inducing a cascade of antioxidant responses [40]. In food crops, only about 1% of the total O_2_ allocated to aerobic metabolism is utilized in ROS generation [41]. Increased activity of antioxidant enzymes and antioxidants can alleviate various oxidative stresses caused by environmental issues [42,43]. In this study, although elevated Cd accumulation in root organelles correlated with increased MDA content, FA application simultaneously enhanced SOD and POD activities in roots with the exception of CAT activity under Cd exposure. With regard to shoots, the response to increased antioxidant enzymes activity was less pronounced. It has been shown that SOD activity can be induced by FA application in abiotic-stressed wheat [44], consistent with our findings. This suggested FA mitigates oxidative stress through activation of antioxidant enzymes in roots rather than antioxidant responses in whole plant.

Previous studies have shown that foliar sprays of boron, nanosized selenium and zinc could alleviate Cd toxicity in wheat by coordinately down-regulating the expression of *TaNramp5*, *TaLCT1* and *TaHMA2* to reduce Cd uptake and transport, and up-regulating *TaHMA3* to enhance vacuole sequestration [45,46]. In our study, FA sprays possessed similar effects on Cd transport and accumulation genes expression. FA-induced downregulation of *TaNramp5*, suggesting less Cd uptake by roots. However, the persistent Cd accumulation in FA-treated roots compared to Cd-only stress indicated further activation of Cd retention in roots. Wu et al. (2018) demonstrated that reduced *OsHMA2* gene expression decreased Cd accumulation in rice xylem sap, thereby diminishing Cd translocation to shoot tissues [47]. In this study, FA-induced suppression of the tonoplast efflux transporter *TaHAM2*, provides a mechanistic basis for diminished root-to-shoot Cd translocation. This suppression of efflux activity was coupled with FA-driven upregulation of the vacuolar sequestration gene *TaHAM3*, synergistically enhancing Cd retention in root vacuoles. In addition, enhanced Cd retention in root soluble fractions (predominantly comprising vacuolar components rich in metal-chelating ligands) suggested the promotion of Cd vacuolar sequestration in roots induced by FA application. This aligned with the established function of *OsHMA3* in mediating vacuolar Cd compartmentalization to limit xylem transport in rice [48]. This mechanism likely involves FA-induced glutathione biosynthesis and phytochelatin-mediated Cd chelation within vacuoles [38]. On the other hand, Cd content in cell wall fractions increased after FA application. As has been documented, FA participated in the formation of plant cell walls through the shikimic acid pathway, where ferulate polysaccharide esters were the initiation and nucleation sites for lignification [49]. Moreover, FA in plant cell walls formed ether linkages with lignin through the hydroxyl groups in the aromatic ring and were ester-linked to plant cell wall polysaccharides [50]. These cross-linkages increased the mechanical strength of the cell wall and were conducive to the Cd fixation in cell wall. The *LCT1* gene encoded a transporter protein that is essential for phloem loading of Cd. Gene silencing of *LCT1* decreased Cd transfer from xylem to phloem, thereby reducing Cd concentrations in phloem sap [8]. Given that 98.8% of grain Cd derives from phloem transport in crop plant [51], the FA-induced downregulation of *TaLCT1* in roots, stems and leaves provided molecular evidence for reducing Cd flux to grains. During grain-filling period, FA sprays caused lower grain Cd concentrations than those of CK, eventually decreasing by 66.3% at maturity. This transcriptional suppression directly regulated Cd mobilization through the source-to-sink phloem pathway. The observed differential Cd partitioning between roots and shoots under FA treatment corroborates the hypothesis of organ-specific stress adaptation mechanisms triggered by exogenous FA application [44].

Cd can inhibit the enzymes related to photosynthesis and affect plant growth by altering transpiration, respiration, and stomatal switch, thereby inhibiting crop photosynthesis. The gas exchange parameters of *C*_i_ and *T*_r_ are limiting factors for CO_2_ diffusion and immobilization, which are associated with the activities of CO_2_ immobilized enzymes, ribulose diphosphate carboxylase, and oxygenase [52]. In this study, when exogenous FA application on wheat leaves was conducted, the improvement of *C*_i_, *g*_s_ and *T*_r_ was recorded, and the Cd inhibition on photosynthesis was reduced, thereby enhancing photosynthesis performance. The increase in *P*_n_ value after foliar FA treatment may be attributed to the increase in *g*_s_ and *T*_r_, which accelerates the effective carbon assimilation period of wheat leaves and facilitates the distribution of photosynthetic products. Therefore, FA spraying can protect photosynthetic system and improve photosynthetic efficacy. This provides a novel phytoregulator-based strategy for improving crop photosynthetic performance in heavy metal-contaminated environments. FA, as one of phenolic acids, also played an ecological regulatory role in soil ecosystem. When the FA content increased in the soils, it can inhibit microbial function groups and enzyme activities related to N transformation in soils and thereby decreased the available N content. However, our findings demonstrated that the regulatory potential for safety crop production in Cd contaminated soils by FA rational application should not be ignored. In future, comprehensive field trials should be implemented to decode FA’s spatiotemporal coordination with nutrient amendments and microbial community to optimize crop-specific application protocols.

## 5. Conclusions

Our multiple investigations demonstrated that foliar-applied FA effectively mitigates Cd toxicity in wheat. FA application enhanced root Cd tolerance by promoting Cd subcellular compartmentalization, increasing antioxidant enzyme activities and restricting Cd xylem loading. These adaptations reduced root-to-shoot Cd translocation. During the grain-filling stage, enhanced stomatal conductance further facilitated Cd accumulation in non-edible spike organs through rachides, thereby reducing the accumulation of Cd in the grains. Our findings establish FA foliar application as an efficient agronomic strategy that reconciled wheat safety production with detoxification potential in Cd-contaminated farmlands.

## Figures and Tables

**Table 1:** Biomass accumulation and root-to-shoot ratio in wheat seedlings under different treatments. All data are presented as the mean ± standard deviation of three biological replicates. Different lowercase letters indicate significant differences between treatments (*p* < 0.05).

Treatment	Plant Height (cm)	Shoot Dry Weight (g Plant^−1^)	Root Dry Weight (g Plant^−1^)	Root-to-Shoot Ratio
CK	63.74 ± 2.01 b	0.21 ± 0.01 a	0.035 ± 0.001 b	0.16 ± 0.006 e
Cd	53.67 ± 2.41 e	0.15 ± 0.01 c	0.027 ± 0.001 e	0.18 ± 0.002 cd
FA20	67.00 ± 1.24 a	0.23 ± 0.02 a	0.040 ± 0.002 a	0.17 ± 0.005 d
FA50	66.67 ± 1.37 a	0.22 ± 0.01 a	0.034 ± 0.002 b	0.15 ± 0.002 f
FA100	60.83 ± 1.22 c	0.18 ± 0.01 b	0.039 ± 0.002 a	0.21 ± 0.004 bc
Cd + FA20	56.17 ± 1.52 d	0.14 ± 0.01 c	0.032 ± 0.001 c	0.22 ± 0.005 ab
Cd + FA50	57.67 ± 0.79 d	0.14 ± 0.03 c	0.030 ± 0.001 d	0.21 ± 0.001 b
Cd + FA100	51.17 ± 2.13 e	0.13 ± 0.01 d	0.029 ± 0.002 e	0.23 ± 0.009 a

**Table 2:** Variations in root morphological traits of wheat under different treatments. All data are presented as the mean ± standard deviation of three biological replicates. Different lowercase letters indicate significant differences between treatments (*p* < 0.05).

Treatment	Total Root Length (cm Plant^−1^)	Root Surface Area (cm^2^ Plant^−1^)	Root Volume (cm^3^ Plant^−1^)	Root Average Diameter (mm)	Root Tips (Tips Plant^−1^)
CK	655.12 ± 42.90 ab	60.29 ± 8.20 c	0.96 ± 0.09 e	0.41 ± 0.05 e	488.33 ± 29.50 e
Cd	538.16 ± 6.21 c	61.86 ± 1.75 c	0.83 ± 0.06 f	0.43 ± 0.01 d	444.50 ± 8.39 f
FA20	681.55 ± 7.76 a	84.29 ± 2.75 b	1.43 ± 0.06 bc	0.46 ± 0.02 cd	551.67 ± 63.69 bcde
FA50	559.61 ± 33.94 c	79.69 ± 10.06 b	1.57 ± 0.32 abc	0.47 ± 0.05 bcd	521.50 ± 79.98 bcd
FA100	708.72 ± 75.18 a	97.55 ± 2.32 a	1.76 ± 0.11 a	0.44 ± 0.01 cd	758.50 ± 38.50 a
Cd + FA20	598.74 ± 55.22 bc	82.82 ± 6.55 b	1.65 ± 0.15 ab	0.48 ± 0.01 abc	529.67 ± 63.31 b
Cd + FA50	420.03 ± 31.01 d	65.73 ± 0.73 c	1.38 ± 0.05 cd	0.52 ± 0.03 ab	577.33 ± 26.16 bc
Cd + FA100	363.10 ± 27.07 e	57.78 ± 1.97 e	1.21 ± 0.03 de	0.53 ± 0.03 a	403.33 ± 31.27 f

**Table 3:** Transport factors in various tissues of wheat plants between controlled and ferulic acid treatments under field experiment conditions during the grain-filling period. All data are presented as the mean ± standard deviation of three biological replicates. The asterisk indicates significant differences between treatments at *p* < 0.05.

Translocation Factor		Day After Anthesis				
		7 d	14 d	21 d	28 d	40 d
TF_root/stem_	CK	1.12 ± 0.01	0.29 ± 0.01	0.28 ± 0.01	0.36 ± 0.06	0.25 ± 0.01
	FA	0.36 ±0.07 *	0.25 ± 0.02 *	0.33 ± 0.05	0.17 ± 0.02 *	0.13 ± 0.04 *
TF_stem/leaf_	CK	3.10 ± 0.41	0.99 ± 0.08	2.10 ± 0.13	0.88 ± 0.46	2.64 ± 0.44
	FA	2.15 ± 0.38 *	3.15 ± 0.40 *	3.00 ± 0.17 *	1.59 ± 0.49 *	1.44 ± 0.14 *
TF_stem/rachis_	CK	0.11 ± 0.04	0.63 ± 0.12	1.33 ± 0.09	1.01 ± 0.17	2.17 ± 0.25
	FA	2.75 ± 0.44 *	1.30 ± 0.32 *	0.77 ± 0.05 *	0.46 ± 0.02 *	0.26 ± 0.08 *
TF_leaf/rachis_	CK	0.33 ± 0.01	0.63 ± 0.05	0.64 ± 0.03	1.89 ± 0.97	0.76 ± 0.01
	FA	1.32 ± 0.16 *	0.39 ± 0.09 *	0.24 ± 0.05 *	0.34 ± 0.08 *	0.21 ± 0.05 *
TF_rachis/gain_	CK	1.81 ± 0.57	1.16 ± 0.17	0.55 ± 0.04	1.46 ± 0.41	1.01 ± 0.12
	FA	0.20 ± 0.02 *	0.64 ± 0.05 *	0.52 ± 0.06	0.55 ± 0.39 *	0.48 ± 0.03 *

## Data Availability

Data are contained within the article.

## Appendix A

**Table A1:** Specific primers for qPCR.

Primer Names	Primer Sequences for the qRT-PCR Analysis	
	Forward Primer 5′→3′	Reverse Primer 5′→3′
*TaNramp5*	TCTGGGTGATTCTGATTGGC	GGCTTTGGATACTCGGTCTT
*TaHAM2*	GGGCATCCGCTTATTTGG	TTCCACTGCCTTTCTCCCTC
*TaHAM3*	CCGTCTCTCAGTCCCGTATTG	GGAGCCACCTGAAGGAAGAC
*TaLCT1*	TGTGCTGTTTGGTTGGTCCT	CCGATTGGCGACCTTCGATA
*TaActin*	AAGGCTGTTGGCAAGGTG	GTGGTCGTTCAGAGCAATCC
